# Consumption of a Beverage Containing Aspartame and Acesulfame K for Two Weeks Does Not Adversely Influence Glucose Metabolism in Adult Males and Females: A Randomized Crossover Study

**DOI:** 10.3390/ijerph17239049

**Published:** 2020-12-04

**Authors:** Yoona Kim, Jennifer B. Keogh, Peter M. Clifton

**Affiliations:** 1 Department of Food and Nutrition, Institute of Agriculture and Life Science, Gyeongsang National University, Jinju 52828, Korea; yoona.kim@gnu.ac.kr; 2 UniSA Clinical and Health Sciences, University of South Australia, Adelaide, SA 5000, Australia; Jennifer.Keogh@unisa.edu.au

**Keywords:** artificial sweetener, glucose, insulin, continuous glucose monitoring

## Abstract

There is an association between the consumption of artificial sweeteners and Type 2 diabetes in cohort studies, but intervention studies do not show a clear elevation of blood glucose after the use of artificial sweeteners. The objective of this study was to examine whether two commonly used artificial sweeteners had an adverse effect on glucose control in normal-weight subjects, and in overweight and obese subjects when consumed for 2 weeks. In the study, 39 healthy subjects (body-mass index, kg/m^2^) (18–45) without Type 2 diabetes with an age of 18–75 years were randomly assigned to 0.6 L/day of an artificially sweetened soft drink containing acesulfame K (950) and aspartame (951) or 0.6 L/day of mineral water for 2 weeks each in a crossover study. There was a 4 week washout period with no drinks consumed. Glucose levels were read by a continuous glucose monitor (CGM) during each 2 week period. A 75 g oral glucose-tolerance test (OGTT) was performed at the beginning and end of each intervention period. Blood samples were collected at baseline, and 1 and 2 h for glucose and insulin. A 2 week intake of artificially sweetened beverage (ASB) did not alter concentrations of fasting glucose and fasting insulin, the area under the curve (AUC) for OGTT glucose and insulin, the incremental area under the curve (iAUC) for OGTT glucose and insulin, the homeostatic model assessment for insulin resistance (HOMA-IR), and the Matsuda index compared with the baseline and with the changes after a 2 week intake of mineral water. Continuous 2 week glucose concentrations were not significantly different after a 2 week intake of ASB compared with a 2 week intake of mineral water. This study found no harmful effect of the artificially sweetened soft drink containing acesulfame K (950) and aspartame (951) on glucose control when consumed for 2 weeks by people without Type 2 diabetes.

## 1. Introduction

In 2019, 463 million individuals aged 20–79 years were globally estimated to have diabetes, and this number is projected to rise to 578 million by 2030 and 700 million by 2045 [1]. In line with increasing diabetes prevalence, the consumption of non-nutritive sweeteners (NNSs) containing no or few calories is becoming common as palatable alternatives to caloric sugars [2,3,4]. The NNS market is growing in the United States [5]. NNS consumption was reported in 25.1% of children and 41.4% of adults, according to the National Health and Nutrition Examination Survey (NHANES) 2009–2012 [5].

Meta-analyses of prospective cohort studies showed NNS consumption was associated with an increased risk of Type 2 diabetes mellitus (T2DM) [6,7,8]. However, reverse causation and confounding by weight status were perhaps an issue [9]. Prospective studies also showed a positive association between NNS consumption, and an increased risk of metabolic syndrome [10,11,12] and weight gain [13,14].

In animal studies, saccharin enhanced sodium/glucose cotransporter 1 (SGLT1) activity and thus elevated glucose-absorption capacity [15]. Moreover, exposure to NNS activated intestinal sweet-taste receptors to upregulate the release of glucose-dependent insulinotropic polypeptide (GIP) from proximal K cells, and glucagon-like peptide-1 and -2 (GLP-1, GLP-2) from more distal L cells [16].

However, human data are much less clear-cut. Systematic reviews [17,18] of randomized controlled trials (RCTs), cohort, case-control, and cross-sectional studies, and case series/reports showed no clear effects of NNS on glucose control.

The aim of this study was to assess whether NNS consumption for 2 weeks alters glucose control in healthy normal and obese individuals. The hypothesis was that the consumption of artificial sweeteners aspartame and acesulfame K for 2 weeks would not influence glucose metabolism in heathy subjects.

## 2. Materials and Methods

### 2.1. Ethical Approval and Registration

This study was approved by the University of South Australia Human Research Ethics Committee, and all study participants gave their written informed consent prior to participating. The trial was registered with the Australian New Zealand Clinical Trials Registry (https://www.anzctr.org.au/) ACTRN 12618000104257. Current link: https://www.anzctr.org.au/Trial/Registration/TrialReview.aspx?id=374094&isClinicalTrial=False. AUD 100 were offered to participants on completion of the study.

### 2.2. Study Participants

Participants were recruited by public advertisement and screened for eligibility by questionnaire only from 2 April 2018 to 1 October 2019. Eligibility criteria were as follows: male or female, aged 18–75 years, body-mass index (BMI) of 18–45 kg/m^2^, no known diabetes or metabolic disorders, not taking medication or supplements that may influence glucose levels, no known allergies/intolerances to the artificial sweeteners, and no artificial-sweetener use (non-nutritive sweeteners) for the 2 previous weeks. Participants had to consume a 600 mL artificially sweetened soft drink daily for 2 weeks and mineral water daily for 2 weeks. Exclusion criteria were weight gain or loss of more than 5 kg over the past 3 months.

**Figure 1:**
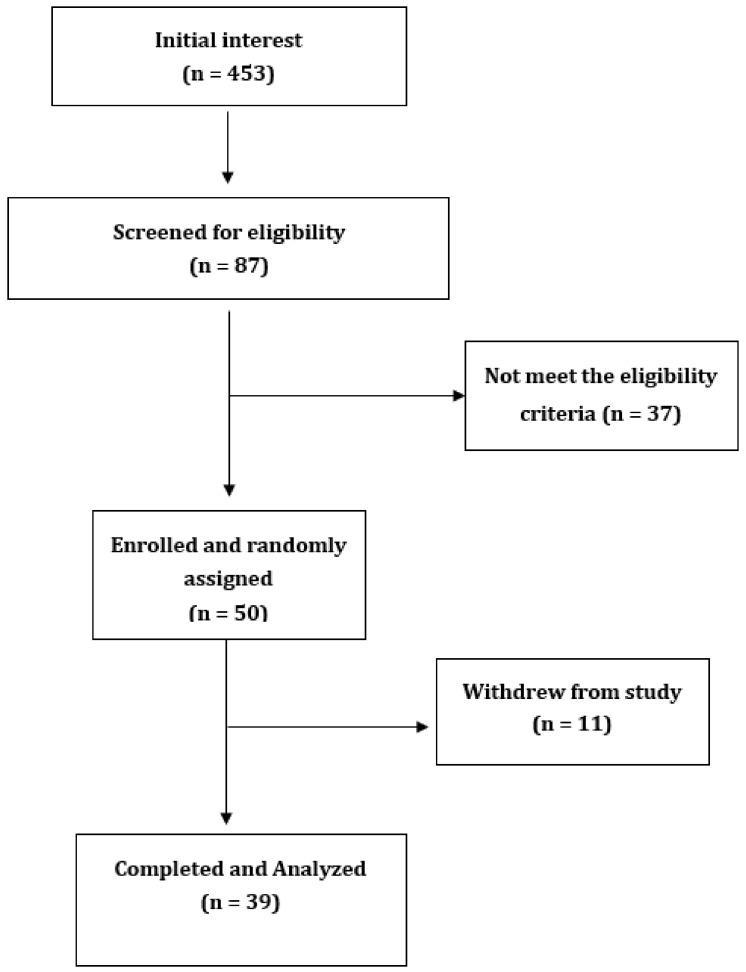
Flowchart of participant recruitment and withdrawal from study.

Initially, a phone-screening interview was conducted with responders who wanted to participate in the study. The interview included completing a short questionnaire to check eligibility, particularly to check for usage of artificial sweeteners and absence of diabetes. Responders who passed the phone screening visited the Sansom Institute for Health Research Clinical Trial facility at the University of South Australia in the morning after an overnight fast. Written informed consent was obtained at the first visit. Details of study recruitment and completion are shown in Figure 1.

### 2.3. Study Design

This was a randomized, crossover trial using commercial home-brand drinks. Drink-assignment order was sequential based on the order of initial recruitment when the questionnaire was originally received in the research office. This assignment was by the office staff and not researchers seeing the volunteers. Participants consumed 0.6 L day of an artificially sweetened beverage (ASB) containing 144 mg/L of aspartame (951) and 211 mg/L of acesulphame K (955), or mineral water for 2 weeks with a 4 week washout period to allow for any changes to the microbiome to revert to normal. This intake was about 5%–6% of the acceptable daily intake. During each 2 week period, subjects wore an Abbott continuous glucose monitor (FreeStyle Libre) 14 day system on the upper arm, which was read at least 4 times/day with a separate reader. Participants had a 75 g oral glucose-tolerance test (OGTT) at the beginning and end of each intervention period, with blood samples taken every 30 min for 2 h for glucose and insulin. Diet was not controlled during each experimental period, but participants were asked to keep their diets relatively constant for each period, minimise eating out, and avoid changes to exercise patterns. Visits took place at the Sansom Clinic with 4 long visits of about 3 h each. A simple survey on activity levels was performed at baseline (sedentary or moderately active); a daily questionnaire asked about changes in activity, diet, and additional soft-drink use, if any.

### 2.4. Measurements during Intervention

An intravenous cannula (BD Nexiva catheters; 20 GA 1.25 IN 1.1 × 32 mm) was inserted at each study visit. Blood samples were collected at 0, 30, 60, 90, 120 min into 2 tubes containing either no additives or sodium fluoride ethylenediaminetetraacetic acid (EDTA). The tube with no additives for serum insulin was kept upright in a tube rack at room temperature for 30 min to ensure complete clot formation and was then placed on ice; the sodium fluoride EDTA tube for plasma glucose was placed immediately on ice until centrifugation and processing. Blood samples were centrifuged at 4000 RPM at 4 °C for 10 min (Universal 32R, Hettich Zentrifugen, Tuttlingen, Germany). Plasma glucose was analysed by an automated spectrophotometric analyser (Konelab 20XTi, Thermo Electron, Waltham, MA, USA). Serum insulin was measured by a ThunderBolt^®^ analyser using Mercodia Insulin ELISA kits (Lot 28563, Uppsala, Sweden).

### 2.5. Glucose Monitoring during Intervention

Insulin sensitivity under fasting conditions was calculated with the homeostasis model assessment of insulin resistance (HOMA-IR) [19] using the following formulas: HOMA-IR = (fasting insulin (μU/mL) × fasting glucose (mmol/L))/22.5.

Insulin sensitivity was assessed from the OGTT by using the methods of Stumvoll et al. [20], calculated as

[0.226 − (0.0032 × BMI) − 0.0000645 × Ins_120_(pmol/L)] − [0.0037 × G_90_(mmol/L)]

where Ins_120_ indicates insulin at 120 min and G_90_ indicates glucose at 90 min; and Matsuda and DeFronzo [21], calculated as

10000 ÷ √ {[G_fasting_ (mg/dL) × Ins_fasting_ (µU/mL)] × [G_meanOGTT_ × Ins_meanOGTT_]}.

Each of these methods previously showed strong correlations with the euglycemic hyperinsulinemic clamp method, which is considered the reference standard for assessing insulin sensitivity [19].

### 2.6. Statistical Analysis

Data were analysed with the use of SPSS V22 (IBM, Chicago, IL, USA). The incremental area for glucose and insulin was calculated using the trapezoidal rule.

The Shapiro–Wilk test, Q–Q plots, and histograms were used to test for normality of distribution. Differences between treatments, and at the beginning and end of each treatment period were tested by ANOVA and paired-sample t tests, respectively. Non-normally distributed variables were log-transformed. Wilcoxon signed-rank nonparametric tests were also used when variables were still skewed after log transformation. Data are presented as the mean ± SD except for skewed variables, which are presented as medians and interquartile ranges. Statistical significance was defined as *p* < 0.05.

## 3. Results

### 3.1. Participant Characteristics

Of 453 people who initially responded to advertising, 87 people replied, and 50 people satisfied the inclusion criteria. Of those, 39 healthy subjects aged 34.5 ± 17 years without T2DM attended the first visit and remained in the study. Figure 1 outlines the recruitment and withdrawal of participants from the study. The baseline characteristics of participants are presented in Table 1. There were 18 participants who had normal weight, 8 were overweight, and 13 were obese. The weight of participants did not significantly change during the study period, as presented in Table 2.

### 3.2. Fasting Glucose, Fasting Insulin, and Area under Curve for Glucose and Insulin

Compared with the baseline values, concentrations of fasting glucose and fasting insulin, HOMA-IR, the area under the curve (AUC) for glucose and insulin, and the incremental area under the curve (iAUC) for glucose and insulin were not significantly different after a 2 week intake of an artificially sweetened beverage (ASB). Moreover, compared with the baseline values, concentrations of fasting glucose and fasting insulin, glucose AUC, glucose iAUC, insulin AUC and insulin iAUC were not significantly different after a 2 week intake of mineral water (MW; Table 2), and changes over the 2 week period were not different between drinks (Table 3). There was no effect of drink order on the results.

### 3.3. Continuous Glucose Concentrations over 2 Weeks

Average continuous-glucose-monitor (CGM) glucose for the ASB period was 5.14 ± 0.74; for the MW period, it was 5.18 ± 0.63. The difference between periods was –0.04 ± 0.61 (N = 39), *p* = 0.7. Changes from the beginning to the end of each period—from Days 1 to 3, to Days 12 to 14—showed a drop of 0.15 ± 0.61 for ASB (*p* = 0.18), and a drop of 0.11 ± 0.65 (*p* = 0.31) for MW. The difference between these changes was not significant, *p* = 0.85 (N = 36). There was no effect of drink order on the results.

## 4. Discussion

This study was designed to examine the effects of 2 week consumption of an ASB containing acesulfame K (950) and aspartame (951) on glucose homeostasis in healthy subjects. No significant effects of ASB were observed on fasting glucose and fasting insulin, glucose AUC and iAUC, and insulin AUC, iAUC, and sensitivity in comparison with baseline values and with any changes seen with mineral water. This finding was consistent with our hypothesis, and it was the same in normal, overweight, and obese people; there was no interaction by weight status.

Very recently, Ahmad et al. [22] observed similar findings to this study, albeit with different sweeteners. The consumption of two types of ASB containing 14% (0.425 g) of the acceptable daily intake (ADI) for aspartame or 20% (0.136 g) of the ADI for sucralose every day for 2 weeks did not significantly change the total OGTT AUC of glucose, insulin, active GLP-1, leptin, and insulin sensitivity compared with baseline values in 17 healthy subjects (24 ± 6.8 years; BMI 22.9 ± 2.5 kg/m^2^) [22].

A few interventions [23,24] were inconsistent with our findings on insulin sensitivity. Sucralose (15% of ADI) consumption for 2 weeks led to a significant incremental change in HOMA-IR 1 week after cessation of the supplement (but not during the intervention), with no differences in fasting concentrations of GLP-1, ghrelin, peptide tyrosine tyrosine (PYY), and leptin in healthy subjects [23]. Sucralose consumption (200 mg/day) for 4 weeks attenuated the acute insulin response after an intravenous glucose-tolerance test (IVGTT), reduced insulin sensitivity (measured by the OGTT Matsuda index), and increased GLP-1 release compared with a placebo in 15 healthy subjects (31.9 ± 10 years; BMI 23.1 ± 3 kg/m^2^) [24].

In our study, glucose concentrations measured by CGM were not different between ASB and MW treatments during the 2 weeks of measurements. In line with these findings, no differences were observed in mean 24 h glucose, iAUC, and total AUC for glucose over 23 h, and 24 h glycaemic variability between 4 types of sweetener beverages containing aspartame, monk fruit, stevia, and sucrose [25]. These CGM findings were in striking contrast to those of Suez et al. 2014 [26], who fed 7 healthy volunteers, who did not normally consume non-nutritional sweeteners, 5 mg/kg of saccharin, i.e., about 120 mg 3 times/day on Days 2–7. The volunteers had daily glucose-tolerance tests assessed using CGM. Four out of 7 had significantly enhanced glucose responses on Days 5–7 compared to Days 1–4. The nonresponders had a different microbiome from the responders, both before and after the saccharin treatment. Animal studies showed similar findings with all artificial sweeteners, including aspartame [26,27,28]. We utilised the findings from the study by Suez et al., given that changes could be seen after 5–7 days of saccharin feeding, by utilising a 2 week study length and a 2 week sweetener-withdrawal period.

Romo-Romo et al. 2016 [18] reviewed 6 chronic feeding studies dating back to 1985 and ranging in duration from 6 to 18 weeks, mostly in people with Type 1 and Type 2 diabetes. They used aspartame, saccharin, sucralose, and stevia, and no effects on glucose metabolism were seen with any tested sweetener. Overall, our study, showing no effects of artificial sweeteners aspartame and acesulfame K on glucose control, was consistent with our recent review indicating that NNS consumption did not differ from water in influencing glucose control [9].

The study was small and relatively short, and different results may be seen after a 3–6 month consumption period. However, in diets of varying carbohydrates and proteins, changes were seen within days in the microbiome [29]. Although the method of randomization was not optimal, it did not involve the researchers, and drink order had no impact on the results. Lastly, volunteers could see their most recent glucose result when they read the sensor. This may have influenced their behaviour, but given that most sugars ranged from 4 to 7, and the volunteers had no prior knowledge of what constituted a normal glucose level, it is not likely to have influenced their eating and drinking behaviour.

## 5. Conclusions

The consumption of an artificially sweetened soft drink containing acesulfame K (950) and aspartame (951) did not alter glucose, insulin, and insulin sensitivity during 2 weeks in individuals without Type 2 diabetes.

## Figures and Tables

**Table 1:** Baseline characteristics of participants ^1^.

Sex (M/F)	13/26
Age (y)	34.5 ± 17
Height (m)	1.7 ± 0.1
Weight (kg)	75.2, 34.8
Body-mass index (BMI; kg/m^2^)	26.1, 9.3
BMI (kg/m^2^)	
Normal	18
Overweight	8
Obese	13
Smoking	
Yes	1
No	38
Alcohol intake	
0–5/week	30
6–10/week	9
Family diabetes history	
Yes	9
No	30
NGT (n)IFG/IGT (n)	33/6
Baseline fasting glucose (mmol/L)	4.9 ± 0.7
Baseline 2 h glucose (mmol/L)	5.5 ± 1.6

^1^ Total participants = 39. Values are mean ± SD except for weight and body-mass index (BMI), which are median and interquartile ranges. M, male; F, female; NGT, normal glucose tolerance; IFG, impaired fasting glucose; IGT, impaired glucose tolerance. Alcohol units are standard 10 g alcohol drinks.

**Table 2:** Effects of artificial sweeteners aspartame and acesulfame K in within-group comparison.

Variables	ASB				MW		
	Baseline	After 2 Weeks	*p* Value	N	Baseline	After 2 Weeks	*p* Value
Fasting glucose (mmol/L)	4.91, 0.82	4.73, 0.72	0.65 ***	35	4.98, 0.7	4.96, 0.7	0.79 ***
Fasting insulin (pmol/L)	8.93, 10.1	9.49, 7.03	0.10 ***	31	8.67, 9.27	8.95, 6.9	0.64 ***
HOMA-IR	1.52, 2.22	1.86, 2.1	0.07 ***	30	2.01, 1.94	1.90, 1.28	0.42 ***
Matsuda index	5.71, 7.22	5.45, 4.97	0.32 **	30	4.67, 5.16	4.94, 4.19	0.47 **
Glucose AUC	13.54 ± 5.88	12.25 ± 2.99	0.14 *	35	13.06, 3.73	12.84, 4.09	0.85 ***
Glucose iAUC	2.27, 4.37	2.51, 3.67	0.09 ***	35	3.21, 4.49	2.59, 3.84	0.45 ***
Insulin AUC	91.9, 84.95	96.3, 84.3	0.35 **	30	105.1, 61.3	94.1, 98.6	0.62 ***
Insulin iAUC	71.3, 52.2	76.7, 74.7	0.27 **	30	95.3, 45.2	78.7, 86.5	0.81 ***
Weight changes	75.2, 34.2	75, 32.6	0.52 ***	39	76.1, 34.2	76.1,33.4	0.69 ***
BMI changes	26.2, 8.9	26.2, 9.4	0.65 **	39	25.9, 9.34	25.4, 9.06	0.47 **

ASB, artificially sweetened beverage; MW, mineral water; HOMA-IR, homeostatic model assessment for insulin resistance; AUC, area under curve; iAUC, incremental area under curve. Normally distributed values are presented as mean ± SD, and *p* values were determined by paired t tests. Non-normally distributed variables (shown as medians and interquartile ranges) were log transformed; *p* values obtained by Wilcoxon signed-rank nonparametric tests, as variables were non-normally distributed after log transformation; * *p* values determined by paired t tests; ** *p* values determined by paired *t* tests after log transformation; *** p values obtained from Wilcoxon signed-rank nonparametric tests.

**Table 3:** Effects of artificial sweeteners aspartame and acesulfame K in between-group comparison.

Variables	ASB	MW	Between-Group Comparison (after ASB vs. after MW)
	Difference at Baseline vs. 2 Weeks	Difference at Baseline vs. 2 Weeks	*p* Value
Fasting glucose (mmol/L)	−0.044, 0.8	0.145, 0.65	0.17
Fasting insulin (pmol/L)	−0.94, 6.29	−0.13, 4.58	0.34
Glucose AUC	0.63, 2.92	0.18, 2.82	0.31
Glucose iAUC	0.39, 3.88	−0.10, 2.9	0.86
Insulin AUC	−8.38 ± 35.6	3.44 ± 40.1	0.17
Insulin iAUC	3.44 ± 40.1	0.07 ± 32.05	0.47

Normally distributed values presented as mean ± SD and *p* values were determined by paired t tests. Non-normally distributed variables (shown as medians and interquartile ranges) were log transformed; *p* values obtained by Wilcoxon signed-rank nonparametric tests as variables were non-normally distributed after log transformation; *p* values for fasting glucose (N = 34), fasting insulin (N = 32), glucose AUC (N = 34), glucose iAUC (N = 33), comparing differences at baseline vs. 2 weeks obtained from Wilcoxon signed-rank nonparametric tests; *p* values for insulin AUC (N = 30) and insulin iAUC (N = 31) comparing differences at baseline vs. 2 weeks obtained from paired *t* tests.
